# Evaluating a Public Health Information Service According to Users’ Socioeconomic Position and Health Status: Protocol for a Cross-Sectional Study

**DOI:** 10.2196/51123

**Published:** 2023-11-24

**Authors:** Mégane Estevez, Sandrine Domecq, Ilaria Montagni, Viviane Ramel

**Affiliations:** 1 Bordeaux University, Inserm, Bordeaux Population Health Research Center, U1219 Bordeaux France

**Keywords:** internet, health information seeking, literacy, social health inequalities, evaluation, digital health, public health, socioeconomic position, health status, user, empowerment, social inequality, mobile phone

## Abstract

**Background:**

The increasing use of information technology in the field of health is supposed to promote users’ empowerment but can also reinforce social inequalities. Some health authorities in various countries have developed mechanisms to offer accurate and relevant information to health care system users, often through health websites. However, the evaluation of these sociotechnical tools is inadequate, particularly with respect to differences and inequalities in use by social groups.

**Objective:**

Our study aims to evaluate the access, understanding, appraisal, and use of the French website *Santé.fr* by users according to their socioeconomic position and perceived health status.

**Methods:**

This cross-sectional study involves the entire French population to which *Santé.fr* is offered. Data will be collected through mixed methods, including a web-based questionnaire for quantitative data and interviews and focus groups for qualitative data. Collected data will cover users’ access, understanding, appraisal, and use of *Santé.fr*, as well as sociodemographic and socioeconomic characteristics, health status, and digital health literacy. A validation of the dimensions of access, understanding, appraisal, and use of *Santé.fr* will be conducted, followed by principal component analysis and ascendant hierarchical classification based on the 2 main components of principal component analysis to characterize homogeneous users’ profiles. Regression models will be used to investigate the relationships between each dimension and socioeconomic position and health status variables. NVivo 11 software (Lumivero) will be used to categorize interviewees’ comments into preidentified themes or themes emerging from the discourse and compare them with the comments of various types of interviewees to understand the factors influencing people’s access, understanding, appraisal, and use of *Santé.fr*.

**Results:**

Recruitment is scheduled to begin in January 2024 and will conclude when the required number of participants is reached. Data collection is expected to be finalized approximately 7 months after recruitment, with the final data analysis programmed to be completed around December 2024.

**Conclusions:**

This study would be the first in France and in Europe to evaluate a public health information service, in this case the *Santé.fr* website (the official website of the French Ministry of Health), according to users’ socioeconomic position and health status. The study could discover issues related to inequalities in access to, and the use of, digital technologies for obtaining health information on the internet. Given that access to health information on the internet is crucial for health decision-making and empowerment, inequalities in access may have subsequent consequences on health inequalities among social categories. Therefore, it is important to ensure that all social categories have access to *Santé.fr*.

**International Registered Report Identifier (IRRID):**

PRR1-10.2196/51123

## Introduction

### Digital Technologies and Health

The health sector is characterized by an exponential use of digital health technology that serves both for care and for coordination among professionals and facilities and for health care system users (eg, health information, appointments scheduling, and communication with caregivers). Digital health technologies are intended to enhance the autonomy of users, including patients and individuals who use these technologies to support the health of their relatives. This implies that users, whether for themselves or for their loved ones, should have access to health information that enables them to better manage health-related concerns.

Although digital health is promising in terms of technology, its increasing use can lead to unintended negative consequences [1-3]. Indeed, digital health technology can mitigate some geographic location–related barriers, but access to, and the use of, these technologies can be influenced by various factors, including socioeconomic position. Geographic location and socioeconomic position remain important factors influencing the use of digital health technologies.

Individuals’ socioeconomic characteristics and their overall health status can affect how different social groups adopt digital health technologies [4]. Consequently, the growing digital divide may worsen existing social health inequalities. The COVID-19 pandemic has accelerated and amplified the digital divide [5]. The lockdown showed that many people were at a loss when faced with the necessity of carrying out web-based procedures. This health, social, and economic crisis has amplified the risks of digital exclusion. Existing digital tools, such as telemedicine and health websites, have been useful in enabling remote medical consultations or keeping up to date with the spread of the virus. By contrast, countless websites are designed according to technical criteria, rather than on the basis of the experience of users in all their diversity [5]. Therefore, it is important to carefully examine the unexpected, undesirable, and negative consequences of digital health to understand its real impact on people and ensure that public health policies address these issues [1-3].

### Social Gradient in Access to Digital Health

Studies highlight a social gradient in the access, use, and appraisal of digital health technologies in other countries [6,7], but data are lacking in France, where individuals belonging to the working classes have less access to computers and are less likely to search for health information on the internet [8].

More recently, using data from the *Baromètre Santé* study conducted between 2010 and 2017, French researchers showed that the profile of health information seekers versus nonseekers has remained consistent over time [9]. Seekers were more likely to be younger, more educated female individuals, with higher household incomes and holding executive positions. In 2017, as in 2014, general health websites remained the top source of information (38.6%), followed by social media and commercial websites, whereas institutional websites ranked as the third source (8.1%) [9]. These authors recommended that health authorities should improve citizens’ digital health literacy and provide reliable and accessible sources of web-based health information.

In response to this issue, some health authorities in different countries have progressively developed mechanisms to provide relevant, accurate, and useful information to health care system users, often through health information websites [10,11]. However, the use of these new sociotechnical tools has been poorly assessed, particularly in terms of differences and inequalities in use among social groups.

### Evaluation of Health Information Websites

The literature includes several studies that have evaluated the quality of health information websites. Some studies have primarily focused on specific information related to particular diseases [12-23] or prevention measures [24-28], whereas other studies have analyzed general health information websites [29-35].

Studies evaluating the quality of health information websites often use automated tools and manual evaluations by professionals. These assessments focus on various criteria, such as readability, the ease of use, reliability, and the quality of information. Tools such as Flesch-Kincaid [14,18,21,22,30,36-41], Flesch Reading Ease [12,18,21,28,30,36-38,41], Simple Measure of Gobbledygook [14,18,21,22,24,30,40], Gunning Fog Index [14,21,22], Coleman-Liau Index [14,21,22], and Automated Readability Index [14,21] measure reading comfort. The Health on the Net Foundation Code of Conduct assesses reliability [17,30,36,42], and the DISCERN tool evaluates health information quality [19,20,28,38,42,43]. The Lida instrument assesses design and content [12,19,37,40], whereas the Clear Communication Index, a tool developed by the Centers for Disease Control and Preventions, evaluates written health materials [32,44]. Some studies also include user perspectives to evaluate website usability and information quality [45,46].

Access to clear and understandable health information is important for informed decision-making, but many health websites are difficult to navigate and read, especially for those with low literacy levels [12,32,37,47]. This can lead to health inequalities [39] and delay the adoption of appropriate health behaviors. Although government websites are usually easier to read [14], improvements are still necessary. Website design and content can also present barriers to use [48]. Few studies focus on the evaluation of a single website, and evaluations often overlook users’ sociodemographic characteristics, health status, and health literacy levels. Therefore, it is important to conduct evaluations that take into account these factors to identify any population groups facing barriers to accessing and understanding health information on these websites.

To make health information websites accessible and user-friendly, it is important to involve users in the evaluation process [32]. Understanding users’ needs and preferences can ensure that the information provided is clear, relevant, and actionable [39]. It is crucial to assess issues related to the accessibility of health information to ensure that all individuals can take advantage of the wealth of health information available on the internet, given the growing focus on patient empowerment in health [48].

### Santé.fr, the Website of the French Public Health Information Service

In France, the *Santé.fr* website is a public service website that provides general health information selected for its quality and usefulness. This website is under the Ministry of Health’s responsibility, and its mission is to provide free information on health care as well as medical and social services to the public. The information is adapted and accessible to people with disabilities and aims to encourage citizen participation in health choices, improve patient management, and simplify the adoption of preventive behaviors.

Santé.fr serves as a reference health website and offers access to resource directories, prevention and health education information services, and more. It is accessible anytime and anywhere, making it a reliable and convenient source of health information.

As *Santé.fr* is an information and support tool for users, it is crucial to track and evaluate its use and identify any disparities among different population groups. Therefore, it is important to explore the experiences and evaluations of *Santé.fr* users to determine to what extent the information provided meets their needs and expectations. The objective is to ensure that *Santé.fr* proves to be a genuine advantage for users and does not exacerbate the existing social inequalities by promoting the use of a tool that is inaccessible or unsuitable for a portion of the population because disparities in the access, understanding, appraisal, and use of digital health technology can worsen social health inequalities, depending on individuals’ social position.

### Objectives of the Study

The main objective of this study was to evaluate the access, understanding, appraisal, and use of *Santé.fr* by users according to their socioeconomic position and health status. Our specific objectives were to (1) determine users’ profiles in terms of the access behavior, understanding, appraisal, and use of *Santé.fr* based on their socioeconomic position and health status; (2) examine the association between users’ socioeconomic position and health status and their access, understanding, appraisal, and use of *Santé.fr*; and (3) determine the reasons why users of *Santé.fr* use this service.

## Methods

### Research Design

The *Évaluation du service public d’information en santé* (the *Santé.fr* website) *selon le statut socio-économique de ses utilisateurs* (evaluation of the public health information service [the *Santé.fr* website] according to the socioeconomic status of users) study is a cross-sectional observational study of the French population using *Santé.fr*. It will use mixed methods, that is, it will combine quantitative and qualitative research methods [49]. The study period will last 1 year so that there will be enough time to collect the necessary quantitative and qualitative data.

### Study Population

The study target population is the entire population to which *Santé.fr* is offered, that is, the general French population. The source population is made up of all health care system users, whether or not they use *Santé.fr*; the respondents to this study; and the study sample. All inhabitants of metropolitan France and the *Départements et régions d’outre-mer et collectivités d’outre-mer* (overseas departments and regions and overseas communities) will be concerned by this study. The quantitative and qualitative study inclusion criterion is as follows: any adult (aged ≥18 y) living in metropolitan France or *Départements et régions d’outre-mer et collectivités d’outre-mer*.

### Recruitment

The quantitative study will include both users and nonusers of *Santé.fr* who visit the website and voluntarily complete the questionnaire. Before participation, all participants will receive comprehensive and adequate information through an information note. *Santé.fr* users are individuals who have previously consulted *Santé.fr* or are visiting it for the first time when completing the questionnaire. Nonusers of *Santé.fr* are individuals who, although aware of this public health information service, have never accessed it before coming across the questionnaire or were not previously familiar with it.

The questionnaire will be self-administered by *Santé.fr* users, using a computer-assisted web interviewing method (ie, completed on the web). An article on *Santé.fr* will be developed to present the research team and the study to participants and to share the external link to the questionnaire. A poster will be designed to encourage users to participate in the study. The article and the questionnaire will be displayed on *Santé.fr* at several navigation levels (eg, thumbnail on the home page, contextual message block, end of an article, and thematic folder) to increase access to the questionnaire. Users connecting to *Santé.fr* will be able to click directly on the poster and reach either the article or the questionnaire. Users will decide whether to participate in the study. A communication plan will be elaborated by the research team to disseminate the study as much as possible.

The qualitative study will include individuals, who may or may not be users of *Santé.fr*, with a variety of profiles, including different sexes, age groups, geographic origins, and socioeconomic positions. Specifically, socioeconomic position will be assessed based on criteria such as education level, occupational category, and income level.

Participants will be recruited through the following channels:

- Relatives or persons in the professional and personal environment of the research team will be requested to identify interested individuals.
- Snowball method: the research team members will ask some of their contacts to identify people they know from different geographic locations and ask them to agree to be contacted by the research team.
- Associations such as Emmaüs Connect, Point Information Mediation Multi Services, and La Mednum will be contacted to identify individuals with digital difficulties. Emmaüs Connect helps individuals experiencing social and digital insecurity to access essential web-based tools. La Mednum helps to develop mediation and digital inclusion programs all over France, as does Point Information Mediation Multi Services.
- Assistance will be requested from the France Assos Santé network, an interassociative organization that represents health system patients and users and defends their interests, to identify patients or users of the health care system.
- Additional resources may be used.

For the quantitative study, a sample size calculation was performed. As we had no information on the proportion of *Santé.fr* users, we calculated the number of participants necessary according to the estimation formula in the case of an expected prevalence of 50% with a risk of error α of 5% and a precision of 5%. A sample of 385 people was deemed necessary. For the qualitative study, we estimate conducting 30 interviews, taking into account feasibility and the need for a wide range of respondent profiles.

### Data Collection

#### Quantitative Study

##### Overview

A questionnaire was developed according to the literature to collect the necessary quantitative and qualitative data to address the objectives. The 5-section questionnaire, described in detail in the following subsections, is available in Multimedia Appendix 1. A feasibility test of the questionnaire will be conducted to ensure that the questions are clear, simple, and understandable.

##### Section 1: Access or Use

This section includes questions to determine whether users or nonusers have the necessary equipment (computer, tablet, smartphone, and internet connection) to access the internet and the general skills needed to use the internet effectively on these devices. The questions also explore their interest in using the internet to find health information and seek to understand how often they use *Santé.fr* for health information. To develop these questions, we used questions from the French *Baromètre Santé* periodic surveys and the Website Quality Evaluation Tool [50]. The *Baromètre Santé* surveys have been conducted since 1992 to better understand French people’s knowledge, behavior, beliefs, and practices concerning health [51].

##### Section 2: Understanding

This section consists of questions related to users’ ability to find and understand health information on *Santé.fr* and their ability to evaluate, in general, health information presented on the internet. These indicators will help determine whether *Santé.fr* is accessible and user friendly. Some items from the eHealth Literacy Scale (eHEALS), discussed in the *eHealth Literacy* subsection, were used [52]. We also used questions from the *Baromètre Santé* periodic surveys [51], the DISCERN tool [53], a systematic literature review by Sun et al [54] on relevant criteria and indicators for evaluating the quality of health information on the internet, and a critical analysis of evaluation criteria for health websites [55].

##### Section 3: Application

This section concerns the appraisal and adoption of *Santé.fr* by users, either for themselves or for relatives. These questions assess whether this website helps users to make informed decisions about their own health. The questions on the intention of users to reuse *Santé.fr* and to recommend it to relatives will allow us to evaluate their satisfaction and to assess their degree of confidence in the provided information. To develop these questions, we were inspired by the technology acceptance model [56] as well as items from the eHealth Impact Questionnaire [57] and items used in the study by Kang and An [33], which evaluated health-related sites for older adults and assessed whether the sites take into account factors that affect older adults’ intention to use them for health information seeking.

##### Section 4: Sociodemographic and Economic Characteristics

This section collects the respondents’ sociodemographic and economic characteristics. The questions cover their indirect geographic origin or native area (and that of their parents), their job status (employed or not) and socioprofessional category or social position, their economic and financial situation (income, origin of income, social benefits, and self-declared financial situation), and their geographic place of residence (postal code and the name of the town of residence). These variables will make it possible to estimate the respondents’ socioeconomic situation. These questions were inspired by data on socioeconomic situations collected from the French Constances study [58].

##### Section 5: Health Status

This section includes questions to evaluate the respondents’ perceived health status, addressing the general, physical, and psychological dimensions of health as well as the presence of an illness or chronic health problem. To develop these questions, we were inspired by the questions from the *Enfants et familles sans logement* (Children and families experiencing homelessness) survey [59] and the periodic *Baromètre Santé* surveys [51].

##### eHealth Literacy

To calculate a digital health literacy score for each respondent and to be able to compare the total scores between *Santé.fr* users and nonusers, the eHEALS items are used [52]: in their original form for nonusers of *Santé.fr* and adapted for users of *Santé.fr* to match the digital tool being evaluated. The corresponding items are presented in Multimedia Appendix 2 and are distributed in the questionnaire’s different sections.

#### Qualitative Study

To explore users’ and nonusers’ perceptions regarding their access, understanding, appraisal, and use of *Santé.fr*, as well as their general use of the internet for searching and using health information, semistructured individual interviews will be conducted. The interview guide will be developed based on the questionnaire used in the quantitative study. Regarding the interview’s organization, an initial telephone contact with the participant will allow us to briefly explain the study to them, obtain their consent to participate in the interview, and set up an appointment for the interview. The participant’s main characteristics will also be collected during this first contact by the interviewer to ensure a wide-range sample. If a participant agrees to participate in the study and has never consulted *Santé.fr*, the interviewer will ask them to navigate through the website before the interview and browse the health topics of interest. On the day of the interview, if the participant has been able to access *Santé.fr*, the interviewer will ask them to describe their overall experience and ask the interview guide questions. If a participant has not been able to access the website, the interviewer will ask them to explain why, which will be considered a result per se.

These interviews will take place via videoconference, telephone, or face-to-face, depending on the participants’ preference, at a time and date of their choice.

Focus groups will also be conducted by the research team. The objective is to enable the participants to provide their reactions to the *Santé.fr* website. Participants will have to navigate the website and share their experience: what they think of *Santé.fr* in general, the difficulties or problems faced, the elements to be improved according to them, their ability to carry out a search and obtain answers to their questions, whether the navigation is easy, and so on. Participants may or may not already know each other and have varied sociodemographic profiles.

### Statistical Analysis

#### Quantitative Analysis

##### Descriptive Analyses

The sociodemographic and socioeconomic characteristics, health status, and digital health literacy score measured by the eHEALS will be described. The variables related to the access, understanding, appraisal, and use of *Santé.fr* will also be presented. Qualitative variables will be analyzed using frequencies and proportions, whereas the digital health literacy scores will be described using mean and SD as well as median and IQR.

A validation of the dimensions of access, understanding, appraisal, and use of *Santé.fr* will be conducted to ensure their reliability and validity. This validation process will involve assessing the internal consistency of items as well as evaluating convergent and discriminant validity. To assess internal consistency, the Cronbach α coefficient will be calculated. In addition, correlation analysis will be conducted to examine the relationships among variables and to evaluate convergent and discriminant validity.

Principal component analysis will be performed for each dimension, using the variables specific to that dimension as active variables. Subsequently, ascendant hierarchical classification will be conducted based on the 2 components of principal component analysis to characterize homogeneous users’ profiles based on their access, understanding, appraisal, and use of *Santé.fr*. The sociodemographic, socioeconomic, and health status variables will be used as illustrative variables to characterize users’ profiles.

##### Analytical Analyses

**Figure 1:**
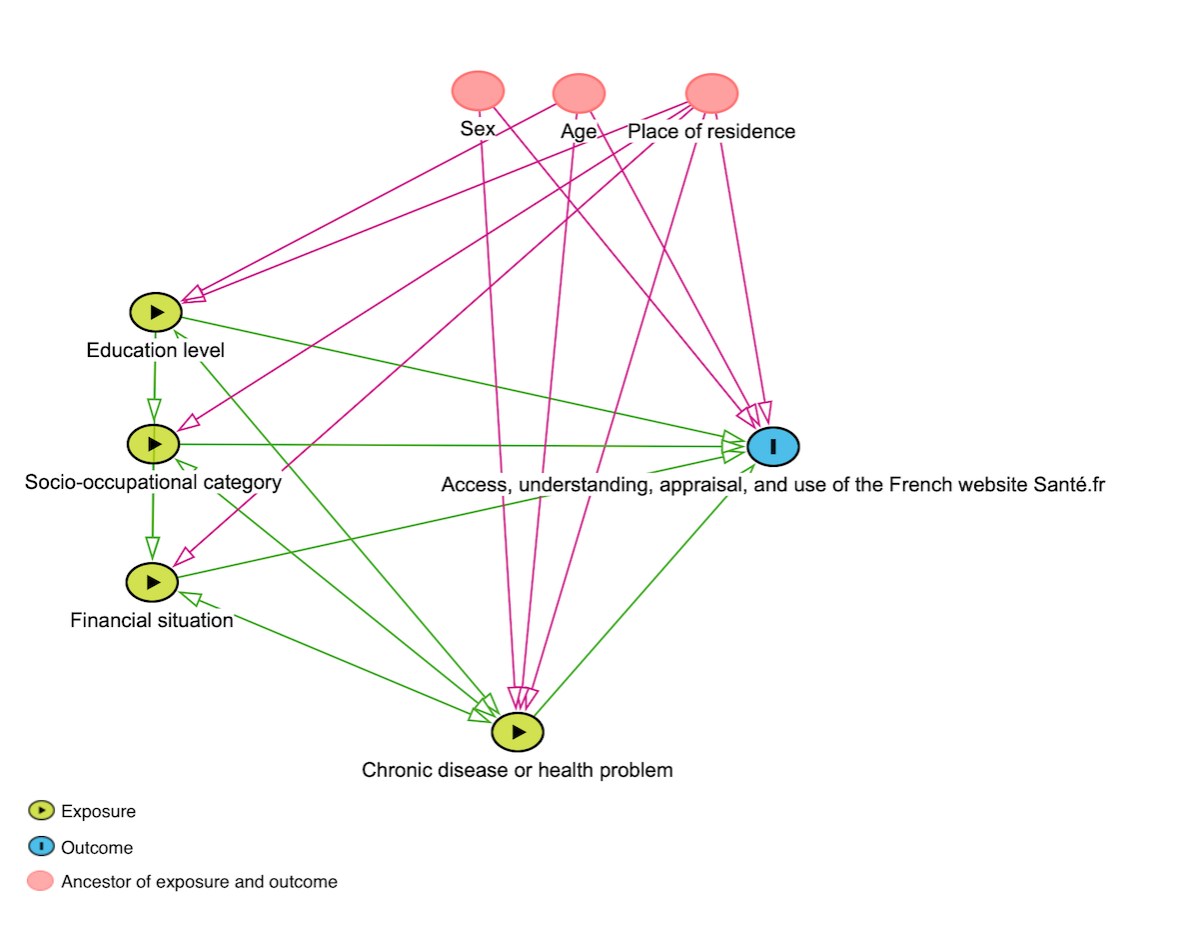
Directed acyclic graph applied to the relationships between socioeconomic position and health status and the access, understanding, appraisal, and use of Santé.fr.

On the basis of the results of the dimension validation in the descriptive part of the statistical analyses, different approaches will be used for the analytical analyses. If the dimension (ie, access, understanding, appraisal, and use of *Santé.fr*) is validated, a quantitative score will be used as a continuous variable. However, if the dimension is not validated, a specific questionnaire variable related to that dimension will be used instead. Depending on the nature of the outcome variable, different regression models will be developed. If the outcome variable is continuous, a linear regression model will be conducted. If the outcome variable is binary, a logistic regression model will be performed. In the case of a variable with >2 categories, a multinomial logistic regression model will be applied. Regardless of the model used, the explanatory variables will include education level, socio-occupational category, and financial situation. Confounding variables such as sex, age, and place of residence will also be included in the analyses to account for potential biases. This approach follows an etiological explanatory perspective, for which a directed acyclic graph will be used (Figure 1).

All quantitative analyses will be conducted using RStudio (version 2023.03.1+446; Posit Software, PBC).

#### Qualitative Analysis

The first step of the qualitative analysis will consist of transcribing all interviews and focus groups. The analysis grid will be constructed from the interview guide and the focus groups and completed inductively based on new items that may appear during the transcripts analysis. Using NVivo 11 software (Lumivero), the interviewees’ comments will be classified within each preidentified theme or themes emerging from the discourse and compared among types of interviewees to understand the reasons why people access, understand, evaluate, and use (or do not) *Santé.fr*. A profile analysis of people with similar responses will be conducted to see whether any profile types emerge.

#### Mixed Analysis

Quantitative and qualitative data will be collected simultaneously using the convergent parallel design approach. The analyses will then be combined to meet the overall objectives of the study.

### Ethical Considerations

The data collection and processing implemented in this research conforms to the *Commission nationale de l’informatique et des libertés* (National Commission of Informatics and Liberties) reference methodology MR-004 (research not involving human persons, studies, or evaluations in the health field).

An information note at the beginning of the questionnaire informs participants about the study, its objectives, its progress, the processing of personal data, and their rights. By completing the questionnaire, the participants implicitly agree to share their data for scientific purposes. For the interviews, the participants will give their oral consent.

Personal data will be processed to analyze the results of the research in relation to its objectives. The data collected will be recorded in a digital file that complies with several data security and confidentiality conditions. The data will be deidentified (ie, anonymized) using the following techniques: we have ensured that the nature of the response modalities is broad enough to prevent the recognition of the person who completed the questionnaire (such as age group and income group); we do not collect nominative data; and we assign a numerical identifier specific to the study to pinpoint the respondent that is known only to the research team.

No compensation will be offered to users for participating in the study.

## Results

Recruitment is scheduled to begin in January 2024 and will conclude when the required number of participants is reached. Data collection is expected to be finalized approximately 7 months after recruitment, with the final data analysis programmed to be completed around December 2024.

## Discussion

### Difficulties and Limitations of the Study

The first difficulty concerning the quantitative part of the study could be linked to the recruitment of participants, given that participation in the study will be on a voluntary basis. Despite our efforts to aim at a wide dissemination at several navigation levels on *Santé.fr* (eg, thumbnail on the home page, contextual message block, end of an article, and thematic folder) to multiply access to the questionnaire, the response rate could prove to be too low to allow statistical analyses to be conducted. For the time being, owing to the different waves of the COVID-19 pandemic, the current pop-in dedicated to screening and vaccination against COVID-19 and appearing on any page after a few minutes of navigation could not be removed and replaced by a message presenting the study. It would therefore be advisable to provide maximum visibility for the study on *Santé.fr*; for example, a pop-in would make it possible to make the study visible to each user and thus increase the chances of response. Moreover, as Santé.fr users prefer to access the website on their mobile phones, if the study does not appear on the home page on mobile phones, the number of respondents could be insufficient can be used.

By contrast, people who feel uncomfortable using the internet could also have difficulties accessing the questionnaire. A lack of motivation or time to complete the questionnaire as well as a low interest in digital health topics and research could decrease the response rate to the study. Compensation for participating in the study could give users a reason to complete the questionnaire.

For the qualitative study too, the lack of sufficiently diverse respondents could be a challenge. In addition, the risk of social desirability bias in the interviewees’ responses to the interviewer could be an issue.

### Strength of the Study

This study would be the first of its kind in France and, more widely, in Europe to focus on the evaluation of a digital health information website according to users’ socioeconomic position.

The study that we intend to set up is thus essential because it will make it possible to fill a gap in the existing literature. More precisely, it will allow us to discover the issues related to inequalities in access to, and the use of, digital technologies to obtain health information. This study will give an insight into the digital divide that accounts for the poor access to these digital health information services for people who do not have access to the necessary material conditions such as digital devices and an internet connection. This study will discover the social inequalities that affect the access, understanding, appraisal, and use of health information found on the internet, particularly among people with lower levels of education, income, and digital health literacy.

As access to health information on the internet is crucial for health decision-making and for people’s empowerment, if inequalities in access are discovered by this study, then they may also be related to inequalities in the health status of social groups. It would therefore be important to ensure that all social groups have equal access to *Santé.fr*, a quality digital health information service offering reliable health information.

With regard to our research subject, using mixed methods is a relevant choice because it will allow us to combine quantitative methods (questionnaire) and qualitative methods (interviews and focus groups). The quantitative methods will make it possible to obtain an overview of access to *Santé.fr* and to measure specific indicators (access, understanding, appraisal, and use) that constitute the digital health literacy determinants. The combination of these 2 methods will give a complete and more accurate view of this evaluation through quantitative results and user experiences that may be influenced by users’ socioeconomic position. Reasons for the use or lack of use can be obtained through the qualitative component of the study.

### Directions for Future Research

This study could serve as a feasibility study for collecting data on the French population’s habits regarding the use of the internet to access health information, which could be incorporated into general national health surveys for regular monitoring purposes as well as *Santé.fr* follow-up surveys.

## Appendix

Multimedia Appendix 1 Study questionnaire.

Multimedia Appendix 2 Original eHealth Literacy Scale and items adapted to this study.

## Abbreviations

eHEALS: eHealth Literacy Scale
