# Supplementary material for: Evaluating a Public Health Information Service According to Users’ Socioeconomic Position and Health Status: Protocol for a Cross-Sectional Study
Source: JMIR Res Protoc. 2023 Nov 24;12:e51123. doi: 10.2196/51123 (PMC10709792; doi:10.2196/51123)
Supplement: Multimedia Appendix 1 [file resprot_v12i1e51123_app1.docx]

## Multimedia Appendix 1

Questionnaire. ESPI3SE study, France, 2023.

| **Section 1: Your access to *Santé.fr*** | | |
| --- | --- | --- |
| **Questions** | **Answering modalities** | **Controls** |
| Q0 - How did you hear about this study? | *(Multiple answers possible)*   - By connecting to *Santé.fr* - Information found via social networks (LinkedIn, etc.) - Media (newspaper article, etc.) - Word of mouth |  |
| Q1 - Do you have an Internet connection at home? | - Yes, good or very good quality (short loading times, online videos are not restrictive) - Yes, but poor quality (heavy loading times, online videos are of poor quality) - No connection |  |
| Q2 - Do you have access in your household to: | *(Multiple answers possible)*   - A computer - A tablet - A smartphone - None of these devices | If the respondent answered "A computer", "A tablet", or "A smartphone", display Q3. |
| Q3 - Are you comfortable with using these devices? | - Not at all comfortable - Somewhat/rather comfortable - Very/quite comfortable |  |
| Q4 - In/over the last 12 months, have you used the Internet? | - Yes, every day - Yes, several times a week - Yes, a few times a month - Rarely - Never |  |
| Q5 - In the past 12 months, have you used Internet to search for health information or advice? (Information about health problems, diseases, health resources or health professionals, health locations, etc.) | - Yes - No | If the respondent answers "No" to Q5, the questionnaire addresses him or her directly to the "You, the Internet and Health" section, which includes the original eHEALS scale, then to section 4, "Your profile," which includes questions on socio-demographic and economic characteristics, and to section 5, "Your health," which includes questions on health status. |
| Q6 - How important do you think it is to be able to access health information on Internet? | - Not at all important - Somewhat important - Very important |  |
| Q7 - The last time you looked for health information using Internet, what topics did you search? | *(Multiple answers possible)*   - Risks related to sexuality, such as AIDS or sexually transmitted infections - Contraception, avoiding pregnancy methods , pregnancy or motherhood - Nutrition, weight gain or eating disorders - Stress, depression, anxiety - Child health or childhood diseases - Alcohol, tobacco, electronic cigarette, cannabis or other illicit drugs - Occasional health problems (infections, flu, etc.) - Public health news (e.g., Covid-19 health crisis, etc.) - Chronic diseases (diabetes, cancer, cardiovascular disease, etc.) - Vaccination (flu, childhood diseases, infectious diseases, Covid-19, etc.) - Aging, disability, autonomy - Water quality, air quality, pollution |  |
| Q8 - Have you ever consulted *Santé.fr*? | - Yes - This is the first time - No, but I was aware of this general public health information service before - No, I was not aware of this public health information service before | If the respondent answered "This is the first time" in Q8, do not display Q9.  If the respondent answers "Yes" then he/she is considered a *Santé.fr* user.  If the respondent answered "No" to Q8, the questionnaire sends him/her  directly to the "You, the Internet and Health" section, which includes the original eHEALS scale, then to section 4, "My profile," which includes questions on socio-demographic and economic characteristics, and to section 5, "Your health," which includes questions on health status. |
| Q9 - How often do you use it? | - Every day - Several times a week - Several times a month - More rarely | If user of *Santé.fr.* |
| Q10 - For what reason(s) did you first access/use it? | *(Multiple answers possible)*   - Out of curiosity - To obtain information about a disease, health problem or symptoms - To obtain information about a treatment/medication - To obtain advice on a treatment/medication - To obtain contact details or information on healthcare professionals, healthcare structures or services - Because *Santé.fr* is an official, reliable site - Other | If user of *Santé.fr.* |
| Q11 - The last time you searched for information on *Santé.fr*, for whom was the search done? | *(Multiple answers possible)*   - For myself - For someone close to me (family member, friend, neighbor, etc.) - Other | If user of *Santé.fr.* |
| Q12 - Generally speaking, when you are on *Santé.fr*, is it easy to navigate the site? (Simplicity of navigation, ease of return to the home page, site map) | - Absolutely - Somewhat - Not at all | If user of *Santé.fr.* |
| **Section 2: Your understanding of the *Santé.fr*** | | |
| Q13 - I know what health resources are available on *Santé.fr* | - Strongly agree - Agree - Undecided - Disagree - Strongly disagree | If user of *Santé.fr.*  Item of eHealth Literacy Scale (eHEALS) |
| Q14 - I know where to find helpful health resources on *Santé.fr* | - Strongly agree - Agree - Undecided - Disagree - Strongly disagree | If user of *Santé.fr.*  Item of eHealth Literacy Scale (eHEALS) |
| Q15 - I know how to find helpful health resources on *Santé.fr* | - Strongly agree - Agree - Undecided - Disagree - Strongly disagree | If user of *Santé.fr.*  Item of eHealth Literacy Scale (eHEALS) |
| Q16 - I know how to use *Santé.fr* to find answers to my health questions | - Strongly agree - Agree - Undecided - Disagree - Strongly disagree | If user of *Santé.fr.*  Item of eHealth Literacy Scale (eHEALS) |
| Q17 - The last time you searched for health information using *Santé.fr*, was it easy to find the information you were looking for? | - Absolutely - Somewhat - Somewhat not Why? _____ - Not at all Why? _____ | If user of *Santé.fr.*  Why = option for the *Santé.fr* team, if they are interested to get this information.  If the respondent checked "Somewhat not" or "Not at all" in Q17, free text option that appears "Why?" (but not required to insert text). |
| Q18 - When you last searched *Santé.fr*, did the information you obtained meet your expectations? | - Absolutely - Somewhat - Somewhat not - Not at all | If user of *Santé.fr.* |
| Q19 - Is the information on *Santé.fr* easy to read?  (For example, use of simple, everyday language, non-technical vocabulary, easy-to-read font, clear headings and subheadings in a larger text size than the main text, acceptable font size for each section, etc.) | - Absolutely - Somewhat - Somewhat not - Not at all | If user of *Santé.fr.* |
| Q20 - Do you easily understand the information or advice provided on *Santé.fr*? | - Absolutely - Somewhat - Somewhat not Why? _____ - Not at all Why? _____ | If user of *Santé.fr.*  Why = option for the *Santé.fr* team, if they are interested to get this information.    If the respondent checked "Somewhat not" or "Not at all" in Q20, free text option that appears "Why?" (but not required to insert text). |
| Q21 - What types of content do you look for on *Santé.fr* in general? | *(Multiple answers possible)*   - Risks related to sexuality, such as AIDS or sexually transmitted infections - Contraception, avoiding pregnancy methods, pregnancy or motherhood - Nutrition, weight gain or certain eating disorders - Stress, depression, anxiety - Child health or childhood diseases - Alcohol, tobacco, electronic cigarettes, cannabis or other illicit drugs - Occasional health problems (infections, flu, etc.) - Public health news (e.g., Covid-19 health crisis, etc.) - Chronic diseases (diabetes, cancer, cardiovascular disease, etc.) - Vaccination (flu, childhood diseases, infectious diseases, Covid-19, etc.) - Aging, disability, autonomy - Water quality, air quality, pollution | If user of *Santé.fr.* |
| Q22 - I can tell high quality from low quality health resources on the Internet | - Strongly agree - Agree - Undecided - Disagree - Strongly disagree | If user of *Santé.fr.*  Item of eHealth Literacy Scale (eHEALS) |
| Q23 - I have the skills I need to evaluate the health resources I find on *Santé.fr* | - Strongly agree - Agree - Undecided - Disagree - Strongly disagree | If user of *Santé.fr.*  Item of eHealth Literacy Scale (eHEALS) |
| Q24 - In your opinion, is the content presented on *Santé.fr* credible/reliable/trustworthy? | - Absolutely - Somewhat - Somewhat not - Not at all | If user of *Santé.fr.* |
| Q25 - In your opinion, is the source of the content presented on *Santé.fr* easily identifiable? | - Absolutely - Somewhat - Somewhat not - Not at all | If user of *Santé.fr.* |
| **Section 3: The use of *Santé.fr* for your health** | | |
| Q26 - In general, does *Santé.fr* help you increase your health knowledge? | - Absolutely - Somewhat - Somewhat not - Not at all | If user of *Santé.fr.* |
| Q27 - I feel confident in using information from *Santé.fr* to make health decisions | - Strongly agree - Agree - Undecided - Disagree - Strongly disagree | If user of *Santé.fr.*  Item of eHealth Literacy Scale (eHEALS) |
| Q28 - In general, do you get answers to your questions when you visit *Santé.fr*? | - Absolutely - Somewhat - Somewhat not - Not at all | If user of *Santé.fr.* |
| Q29 - I know how to use the health information I find on *Santé.fr* for my own health | - Strongly agree - Agree - Undecided - Disagree - Strongly disagree | If user of *Santé.fr.*  Item of eHealth Literacy Scale (eHEALS) |
| Q30 - Do you think *Santé.fr* has helped you make decisions about your own health or that of a relative? | - Absolutely - Somewhat - Somewhat not - Not at all - Not concerned | If user of *Santé.fr.* |
| Q31 - Does *Santé.fr* encourage you to take care of your health or that of a relative? | - Absolutely - Somewhat - Somewhat not - Not at all | If user of *Santé.fr.* |
| Q32 - Do you intend to use *Santé.fr* again to find health information? | - Yes - No | If user of *Santé.fr.* |
| Q33 - Do you intend to recommend *Santé.fr* to your friends and family? | - Yes - No | If user of *Santé.fr.* |
| **Section 4: Your profile** | | |
| Q34 - What is the post code and name of your municipality of residence? | Postal Code: _ _ _ _ _  Name of the municipality: ________ | Allow 6 digits maximum & 3 minimum for DROM-COM postal codes). |
| Q35 - Do you live in... | - Urban / suburban area (near a large city, in the suburbs) - Rural area |  |
| Q36 - Do you reside... | - At home - In an institution (nursing home, “EHPAD”, home for the disabled, university residence, other) - At a relative's home (parents, friends, room in a home) - Homeless - Other |  |
| Q37 - Are you: | - A man - A woman - Other / non-binary |  |
| Q38 - What is your age range? | - 18-23 years old - 24-29 years old - 30-39 years old - 40-49 years old - 50-59 years old - 60-69 years old - 70 years and older |  |
| Q39 - What is your family situation? | - In a relationship without children - In a relationship with at least one child - Single parent family - Single - Widow/wife |  |
| Q40 - What is your nationality? | - You are a French citizen born to two French parents - You are French and have at least one foreign parent - You are not French |  |
| Q41 - Do you have difficulty reading French? | - Yes - No |  |
| Q42 - What is the highest degree you have reached? | - No degree - Certificate of primary studies, college certificate, first cycle diploma or an equivalent qualification - Certificate of professional competence, vocational diploma or an equivalent qualification - Vocational or technological baccalaureate - General baccalaureate - Two years of college education - Bachelor’s degree or an equivalent qualification - Master’s degree 1 or an equivalent qualification - Master’s degree 2 or an equivalent qualification - PhD or an equivalent qualification |  |
| Q43 - What is your professional/job situation? | - Employed - Unemployed and not looking for a job - Unemployed or looking for work - Student, apprentice - Retired - Other |  |
| Q44 - In your opinion, your current or past job is closest to which category? | - Farmer - Tradesman, shopkeeper or business owner with fewer than 10 employees - Executive, self-employed or company director with more than 10 employees, teacher in higher education or secondary school - Intermediate occupation (technician, supervisor, elementary school teacher, etc.) - Employee - Worker - I don’t know | Q44 appears only if the respondent answered "Employed", "Unemployed and not looking for a job", "Unemployed or looking for work", "Retired" to Q43. |
| Q45a - Is/was your occupation related to human, animal or public health (e.g. physician, pharmacist, midwife, nurse, caregiver, osteopath, veterinarian, etc.) | - Yes - No |  |
| Q45b - Is your education related to human, animal or public health? (e.g. physician, pharmacist, midwife, nurse, nursing assistant, osteopath, veterinarian, etc.) | - Yes - No | Q45b appears only if the respondent answered "Student, apprentice" in Q43. |
| Q46 - From the list below, check the box that corresponds to your household's net monthly income (including all salaries, social benefits, allowances, rental income, etc.) after social contributions and before taxes: | - Less than 450 € - From 450 € to less than 1000 € - From 1000 € to less than 1500 € - From 1500 € to less than 2100 € - From 2100 € to less than 2800 € - From 2800 € to less than 4200 € - 4200 € or more - I don't know / I don't want to answer |  |
| Q47 - Do you have supplementary health insurance? | - Yes, a mutual insurance company, a complementary insurance company or a provident institution - Yes, supplementary health insurance (formerly known as CMU-C) - No, no complementary health coverage |  |
| Q48 - What is the source of your personal income? | *(Multiple answers possible)*   - Professional income (salaries, etc.) - Unemployment benefits or allowances - Retirement pension(s), survivor's pension(s), pre-retirement allowance(s), minimum old age benefit(s), solidarity allowance for the elderly, etc. - Active solidarity income (RSA), disabled adults' allowance (AAH), personalized autonomy allowance (APA), compensatory allowance for a third party (ACTP) or for professional expenses (ACFP), compensation benefit, disability pension, supplementary disability allowance (ASI), partial permanent disability pension, education allowance for disabled children (AEEH), daily parental presence allowance (AJPP), other family assistance(s) : allowance(s) for the reception of young children (PAJE), family complement, family support allowance - Housing assistance (housing allowance, personalized housing assistance) - Daily sickness benefit(s) in case of illness, occupational disease, work accident, maternity, paternity or adoption - Income from capital (rent, dividends, etc.) - Alimony or child support - Regular assistance from family or friends - Scholarship, aid(s) to finance studies - No personal income or only spouse's income |  |
| Q49 - Given your household income, currently, would you say that financially: | - You are comfortable/well-off - It is sufficient - It is difficult |  |
| **Section 5: Your health** | | |
| Q50 - In general, would you say you feel: | - Very well surrounded - Somewhat surrounded - Somewhat lonely - Very lonely |  |
| Q51 - How would you rate your overall health? | - Very good - Good - Fair - Poor - Very poor |  |
| Q52 - How do you rate your physical health? | - Very good - Good - Fair - Poor - Very poor |  |
| Q53 - How do you rate your moral, psychological and emotional health? | - Very good - Good - Fair - Poor - Very poor |  |
| Q54 - Do you currently have a chronic illness or health problem? | - Yes - No | If "Yes" display Q55. |
| Q55 - If yes, what disease(s)/problem(s) are involved? | *(Multiple answers possible)*   - Cardiovascular disorder (arrhythmia, tachycardia, hypertension, etc.) - Cancer (any type) - Diabetes (any type) - Kidney failure or other chronic kidney disease - HIV or other immunity disorder - Mental health disorder (anxiety, depressive disorders, etc.) - Neurological disorder (epilepsy, multiple sclerosis, migraine, etc.) - Severe liver disease (cirrhosis, NASH, hepatitis, etc.) - Chronic insomnia disorder (insomnia, obstructive sleep apnea syndrome, etc.) - Gastrointestinal disorder (functional dyspepsia, irritable bowel syndrome, reflux hypersensitivity, etc.) - Respiratory disorder (respiratory failure, asthma, chronic bronchitis, etc.) - Musculoskeletal or osteoarticular disorder - Physical disability - Gynecology disorder - Post-COVID-19 syndrome - Other |  |
| **You, the Internet and Health**  Original eHealth Literacy Scale (eHEALS) | | |
| I know what health resources are available on the Internet | - Strongly agree - Agree - Undecided - Disagree - Strongly disagree |  |
| I know where to find helpful health resources on the Internet | - Strongly agree - Agree - Undecided - Disagree - Strongly disagree |  |
| I know how to find helpful health resources on the Internet | - Strongly agree - Agree - Undecided - Disagree - Strongly disagree |  |
| I know how to use the Internet to answer my health questions | - Strongly agree - Agree - Undecided - Disagree - Strongly disagree |  |
| I know how to use the health information I find on the Internet to help me | - Strongly agree - Agree - Undecided - Disagree - Strongly disagree |  |
| I have the skills I need to evaluate the health resources I find on the Internet | - Strongly agree - Agree - Undecided - Disagree - Strongly disagree |  |
| I can tell high quality from low quality health resources on the Internet | - Strongly agree - Agree - Undecided - Disagree - Strongly disagree |  |
| I feel confident in using information from the Internet to make health decisions | - Strongly agree - Agree - Undecided - Disagree - Strongly disagree |  |
