# Supplementary material for: Evaluating a Public Health Information Service According to Users’ Socioeconomic Position and Health Status: Protocol for a Cross-Sectional Study
Source: JMIR Res Protoc. 2023 Nov 24;12:e51123. doi: 10.2196/51123 (PMC10709792; doi:10.2196/51123)
Supplement: Multimedia Appendix 2 [file resprot_v12i1e51123_app2.docx]

## Multimedia Appendix 2

Original eHealth Literacy Scale and items adapted to the *Santé.fr*. ESPI3SE study, France, 2023.

| **Original eHealth Literacy Scale (eHEALS)** | **eHealth Literacy Scale adapted to *Santé.fr*** |
| --- | --- |
| I know what health resources are available on the Internet | I know what health resources are available on *Santé.fr* |
| I know where to find helpful health resources on the Internet | I know where to find helpful health resources on *Santé.fr* |
| I know how to find helpful health resources on the Internet | I know how to find helpful health resources on *Santé.fr* |
| I know how to use the Internet to answer my health questions | I know how to use *Santé.fr* to answer my health questions |
| I know how to use the health information I find on the Internet to help me | I know how to use the health information I find on *Santé.fr* to help me |
| I have the skills I need to evaluate the health resources I find on the Internet | I have the skills I need to evaluate the health resources I find on *Santé.fr* |
| I can tell high quality from low quality health resources on the Internet | I can tell high quality from low quality health resources on the Internet |
| I feel confident in using information from the Internet to make health decisions | I feel confident in using information from *Santé.fr* to make health decisions |
